# Two distinct clinical progressions of P67phox-deficient CGD, both commencing with cervical lymphadenitis

**DOI:** 10.1186/s13052-024-01813-8

**Published:** 2024-11-05

**Authors:** Lili Dong, Lei Zhang, Chunna Xu, Mingfa Guo, Yu Tang, Yuelin Shen

**Affiliations:** 1 Respiratory Department, Children’s Hospital Affiliated to Zhengzhou University, Henan Children’s Hospital, Zhengzhou Children’s Hospital, NO.33, Longhu Outer Ring East Road, Zhengdong New District, Zhengzhou, 450018 China; 2 Henan Medical Key Laboratory of Pediatric Hematology, Children’s Hospital Affiliated to Zhengzhou University, Henan Children’s Hospital, Zhengzhou Children’s Hospital, Zhengzhou, 450018 China; 3 Respiratory Department II, National Clinical Research Center for Respiratory Diseases, Beijing Children’s Hospital, Capital Medical University, National Center for Children’s Health, Beijing, NO.56, Nanlishi Road, 100045 China

**Keywords:** Chronic granulomatous disease, Phenotype, NCF2 gene, Variant, Chinese

## Abstract

We present two rare cases of p67phox-deficient chronic granulomatous disease (CGD) caused by compound heterozygous mutations in the *NCF2* gene. They developed cervical lymphadenitis as the initial manifestation of CGD but had distinct clinical progressions. Patient 1 presented with aspergillous meningitis, an extremely rare manifestation of neurological involvement in CGD, which has not been reported before. Patient 2 presented with non-infectious inflammatory lymphadenitis is also very rare and has not been reported previously. These cases emphasize the importance of considering p67phox-deficient CGD in children with late-onset invasive fungal infections and non-infectious inflammatory lesions. Additionally, we also reviewed previous reports of Chinese patients with P67phox-Deficient CGD. Our objective is to raise awareness about the clinical, diagnostic, and genetic characteristics of P67phox-deficient CGD in China, to reduce misdiagnosis and improve the management and prognosis of the disease.

**Supplementary Information:**

The online version contains supplementary material available at 10.1186/s13052-024-01813-8.

## Main text

To the Editor,

Chronic granulomatous disease (CGD) is a rare inherited immunodeficiency disorder with an estimated prevalence of 1:250,000 to 1:200,000 live births. It results from defects in the components of the nicotinamide adenine dinucleotide phosphate (NADPH) oxidase enzyme in phagocytes [1]. Specifically, mutations in *NCF2*, which encodes the cytosolic factor p67phox, cause one of the rarest forms of autosomal recessive CGD, accounting for 7% of all cases [2]. Patients with CGD have impaired production of reactive oxygen species that are essential for bacterial and fungal eradication, predisposing them to severe, recurrent infections across multiple organs. Pulmonary involvement, particularly Aspergillus pneumonia, is the most common and lethal manifestation [3]. Some patients also experience granuloma formation due to an excessive inflammatory response [3]. This study details two distinct clinical progressions of p67phox-deficient CGD, both commencing with cervical lymphadenitis.

**Fig. 1:**
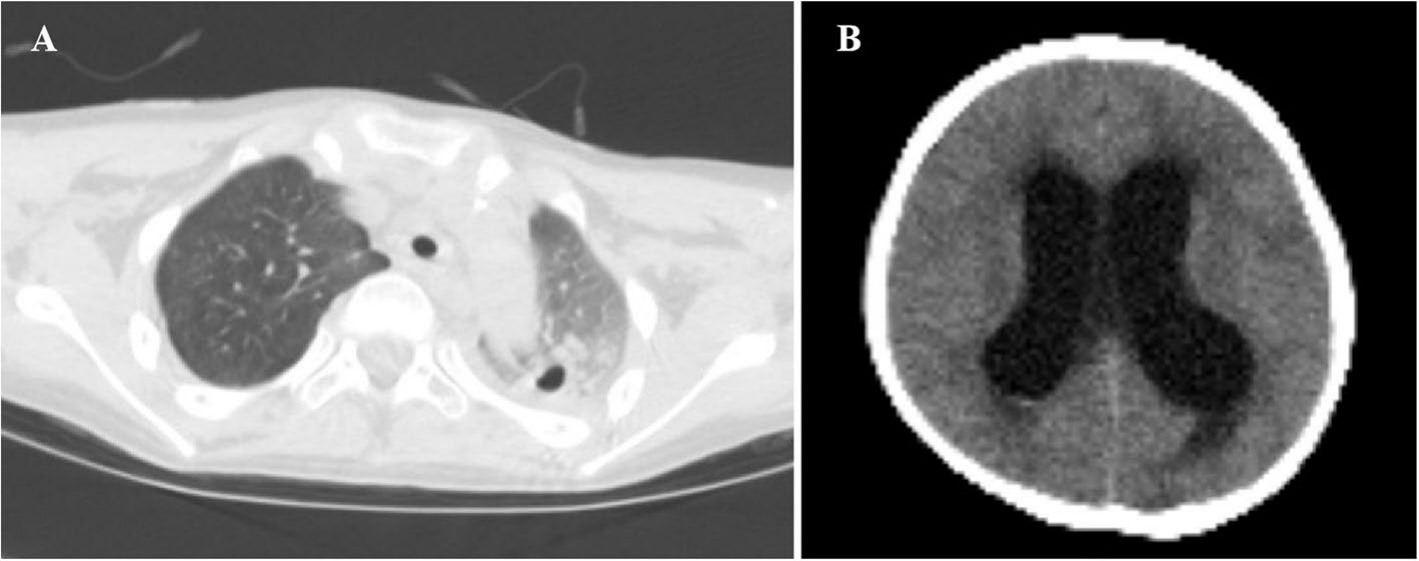
CT scans of Patient 1 with p67phox-deficient CGD, demonstrating cavitary pneumonia (Panel **A**) and communicating hydrocephalus (Panel **B**)

**Fig. 2:**
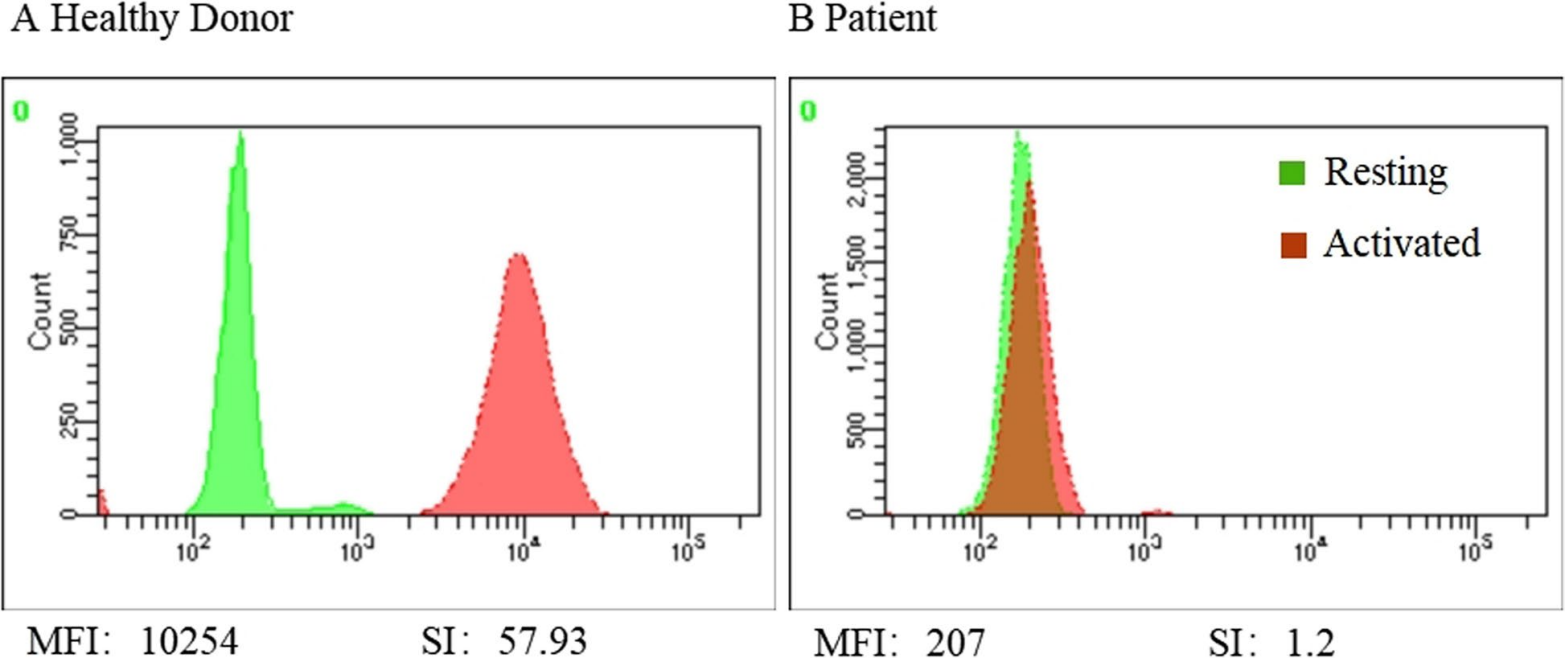
Respiratory burst assay results in Patient 1 with p67phox-deficient CGD, which exhibited a reduced activation rate of 4.5%

Patient 1, an 8-year-old male, presented with cervical lymphadenitis, with the largest lymph node measuring 2.7 cm × 1.0 cm. Subsequently, he developed severe pulmonary aspergillosis with cavitation (Fig. 1A), receiving a month of antifungal treatment before self-withdrawal. At age 9, he exhibited fever and headache, and a lumbar puncture indicated increased intracranial pressure and cerebrospinal fluid (CSF) leukocytosis, with a predominance of polymorphonuclear cells. Aspergillus fumigatus was consistently identified in the CSF via next-generation sequencing (NGS). Magnetic resonance imaging scans showed multiple meningeal and nerve root enhancements, leading to a central nervous system (CNS) aspergillosis diagnosis, treated with intravenous voriconazole and amphotericin B. At age 10, a head computed tomography (CT) scan revealed communicating hydrocephalus, managed with peritoneal CSF shunt surgery. At age 11, electroencephalogram patterns suggested secondary epilepsy, treated with levetiracetam. By age 12, immunologic tests revealed normal levels of immunoglobulins and lymphocyte subsets, but a dihydrorhodamine test resulted pathological, as the neutrophils showed impaired capacity to activate the NADPH oxidase cascade (Fig. 2B). Genetic testing revealed compound heterozygous *NCF2* variants c.304 C > T(p.Arg102X)/c.323 A > G (p.Asp108Gly), the latter being an unreported novel variant. The patient was finally diagnosed with p67phox-deficient CGD. He received long-term oral trimethoprim-sulfamethoxazole (TMP-SMX) and itraconazole for CGD prophylaxis, and is currently on the waiting list for hematopoietic stem cell transplantation (HSCT).

Patient 2, a 3-year-old female, presented with a 6-day fever and was hospitalized. She exhibited bilateral, non-tender, enlarged cervical lymph nodes, with the largest measuring 2.5 cm × 1.0 cm. Laboratory tests showed elevated immunoglobulins and complement proteins, but normal lymphocyte subsets. Despite negative cultures from blood, sputum, bone marrow, and CSF, she had elevated white blood cells (17.2 × 10^9^/L), C-reactive protein (23.5 mg/L), interleukin-6 (163.5 pg/ml), and erythrocyte sedimentation rate (98 mm/h). Additionally, there were minor elevations in anti-cyclic citrullinated peptide antibody and rheumatoid factors. The chest CT scan showed no abnormalities. Antibiotics were ineffective. A cervical lymph node biopsy indicated inflammatory proliferation with lymphocytic infiltration. Genetic testing revealed compound heterozygous NCF2 variants c.1180T > G (p.Tyr394Asp) and c.304 C > T (p.Arg102X). Intravenous methylprednisolone (2 mg/kg) resolved the fever within three days, with subsequent dose reduction over three weeks. The patient was discharged after 28 days, asymptomatic under TMP-SMX and itraconazole prophylaxis, and remained so at 6-month follow-up. The immunological evaluations of two Chinese children with p67phox-deficient CGD are shown in Supplementary Table 1.

Lymphadenitis affects 50% of CGD patients globally [4]. Causative pathogens include *Staphylococcus aureus*, *Burkholderia cepacia complex*, *Serratia marcescens*, *Nocardia spp*., *Mycobacterium tuberculosis*, *Bacillus Calmette-Guérin* and *Aspergillus spp*. *Aspergillus spp.* (mainly *Aspergillus fumigatus* or *nidulans*) leads to a subacute onset, chronic progression, and episodic exacerbations and remissions, with potential hematogenous spread to the brain or liver. CNS involvement is rare in patients with CGD, occurring in only 4–5% of cases [5]. Cerebral aspergillosis associated with CGD typically manifests as aspergillous abscesses/aspergillomas [6]. Diagnosis is mainly based on histopathological examination. The CSF may show minimal microbiologic and biochemical changes. Only Tsuge et al. reported a case of a 3-year-old boy with X-linked CGD who developed fungal meningitis diagnosed by polymerase chain reaction test of CSF, but the fungal species was not identified [7]. However, NGS technology has enhanced pathogen detection. Patient 1 exhibited aspergillous meningitis, an exceedingly rare neurological presentation in CGD, which has not been reported before. The diagnosis was substantiated through CSF cytology and NGS.

Non-infectious inflammatory complications, such as granuloma formation, systemic inflammation, and autoimmunity, are prevalent in p67phox-deficient CGD [8, 9]. However, the most commonly affected sites are the gastrointestinal tract and lungs [10]. Patient 2 exhibited cervical lymphadenitis without respiratory symptoms. All microbiological tests were negative, but inflammatory markers were persistently elevated. Antibiotics were ineffective, but steroids alleviated inflammation. This indicated that the lymphadenitis was a non-infectious inflammatory complication of CGD. Non-infectious inflammatory lymphadenitis is exceedingly rare and has not been previously reported.

In China, the limited awareness and diagnostic capabilities for CGD frequently result in misdiagnosis, underdiagnosis, and delayed diagnosis. For instance, Patient 1 experienced a four-year delay from the onset of symptoms to the diagnosis of CGD. Such delays can lead to severe consequences, including recurrent and severe infections that complicate treatment. Furthermore, delayed diagnosis may cause patients to lose valuable time before they can be enrolled for HSCT or miss the optimal window for this treatment, thereby adversely affecting long-term survival and quality of life.

According to previous reports, a total of 27 cases of Chinese p67phox-deficient CGD have been reported, accounting for 4.9% of the NCF2 variants in Chinese patients (Table 1), below the global 7% prevalence [3]. The reasons for this discrepancy may include genetic diversity, underdiagnosis, or reporting bias. Notably, both patients in our study had the NCF2 variant c.304 C > T(p.Arg102X), a common cause of p67phox-deficient CGD globally. This nonsense variant truncates peptide synthesis, impairing protein function, and is found in diverse populations, including North Africa, Hispanic, Israeli, Turkish, and Iranian individuals. Clinical manifestations linked to c.304 C > T(p.Arg102X) include Serratia marcescens pneumonia, BCGitis, lymphadenitis, abscesses, and osteomyelitis. This report uniquely identifies disseminated aspergillosis across multiple organs associated with c.304 C > T(p.Arg102X). Patient 1 also harbored the novel *NCF2* variant c.323 A > G(p.Asp108Gly), predicted as pathogenic by tools like SIFT, Polyphen-2, Mutation Taster, and GERP+. Given the clinical presentation and compromised NADPH oxidase activity, we propose the compound heterozygous variants c.304 C > T(p.Arg102X)/c.323 A > G(p.D108G) as the cause of CGD in Patient 1. Patient 2 also possessed the high-frequency variant c.304 C > T(p.Arg102X) and the ethnically specific c.1180T > G(p.Tyr394Asp), reported exclusively in East Asians [11]. The clinical manifestations linked to c.1180T > G(p.Tyr394Asp) remain uncertain, with only pulmonary aspergillosis documented.

**Table 1:** Previous reports of Chinese patients with P67phox-deficient CGD

Ref.	Total CGD cases	P67phox-Deficient CGD cases			
		No. of Cases	Nucleotide change	Region	Genotype
Wu et al., 2017 [4]	48	1	c.550 C > T	Exon 1	Homozygous
Liu et al., 2018 [12]	1	1	c.233G > A	Exon 2	Homozygous
Gao et al., 2019 [11]	147	4	c.196 C > T/c.304 C > T	Exon 1	Compound heterozygous
			c.1180T > G	Exon 13	Homozygous
			c.304 C > T/c.1180T > G	Exon 1/Exon13	Compound heterozygous
			c.172_174delAAG/c.1180T > G	Exon 1/Exon13	Compound heterozygous
Wang et al., 2019 [13]	97	2	c.304 C > T	Exon 3	Homozygous
			c.233G > A	Exon 2	Homozygous
Li et al., 2019 [14]	22	1	c.304 C > T	Exon 1	Homozygous
Guo et al., 2020 [15]	4	1	c.1180T > G/c.1179-1G > C	Exon 13/Exon13	Compound heterozygous
Liu et al., 2021 [16]	139	9	No Data Available		
Shen et al., 2021 [17]	1	1	c.196_197insA/c.1180T > G	Exon 1/Exon13	Compound heterozygous
Sun et al., 2022 [18]	85	5	No Data Available		
Current report	2	2	c.304 C > T/c.323 A > G	Exon 3/Exon 3	Compound heterozygous
			c.304 C > T/c.1180T > G	Exon 3/Exon 13	Compound heterozygous

These two cases highlight the importance of considering p67phox-deficient CGD in children with late-onset invasive fungal infections and non-infectious inflammatory lesions. Our findings contribute to enhancing awareness of the clinical, diagnostic, and genetic characteristics of p67phox-deficient CGD in China, thereby reducing misdiagnosis and improving disease management and prognosis.

## Supplementary Information

Supplementary Material 1.

## Data Availability

Data sharing is not applicable to this article as no datasets were generated or analysed during the current study.

## Abbreviations

CGD: Chronic granulomatous disease
CNS: Central nervous system
CSF: Cerebrospinal fluid
CT: Computed tomography
HSCT: Hematopoietic stem cell transplantation
NADPH: Nicotinamide adenine dinucleotide phosphate
NGS: Next-generation sequencing
TMP-SMX: Trimethoprim-sulfamethoxazole
