# Supplementary material for: Two distinct clinical progressions of P67phox-deficient CGD, both commencing with cervical lymphadenitis
Source: Ital J Pediatr. 2024 Nov 5;50:234. doi: 10.1186/s13052-024-01813-8 (PMC11539424; doi:10.1186/s13052-024-01813-8)
Supplement: Supplementary file 1 — Supplementary Material 1. [file 13052_2024_1813_MOESM1_ESM.docx]

**Supplementary Table 1. Immunological evaluations of two Chinese children with P67phox-Deficient CGD**

| **Variables** | **Patient 1** | **Patient 2** |
| --- | --- | --- |
| Age of symptom onset, years | 8.0 | 3.0 |
| Age at diagnosis, years | 12.2 | 3.1 |
| Sex | Male | Female |
| WBC, ×10^9^/L | 10.2 | 17.2 |
| Neutrophil percentage, % | 66.4 | 77.1 |
| CRP, mg/L | 4.3 | 23.5 |
| ESR, mm/h | 50.0 | 98.0 |
| PCT, ng/mL | 0.1 | 0.6 |
| IL-6, pg/mL | 3.5 | 163.5 |
| IgG, g/L | 10.4 | 14.8 |
| IgM, g/L | 0.6 | 2.6 |
| IgA, g/L | 2.5 | 2.6 |
| CD3+ T cells, % | 81.0 | 70.7 |
| CD8+ T cells, % | 46.0 | 13.0 |
| CD4+ T cells, % | 33.0 | 52.3 |
| NK cells, % | 11.0 | 4.2 |
| B cells, % | 7.0 | 24.3 |
| DHR test | Positive | ND |

**Abbreviations:** CGD: Chronic granulomatous disease; CRP: C-reactive protein; DHR: Dihydrorhodamine; ESR: Erythrocyte sedimentation rate; IgA: Immunoglobulin A; IgG: Immunoglobulin G; IgM: Immunoglobulin M; IL: Interleukin; NK: Natural killer; PCT: Procalcitonin; WBC: White blood cell
